# Glutathione S-Transferase M1 Gene Polymorphism and Laryngeal Cancer Risk: A Meta-Analysis

**DOI:** 10.1371/journal.pone.0042826

**Published:** 2012-08-10

**Authors:** Xin-Jiang Ying, Pin Dong, Bin Shen, Cheng-Zhi Xu, Hong-Ming Xu, Shu-Wei Zhao

**Affiliations:** 1 Department of Otolaryngology-Head and Neck Surgery, Shanghai First People’s Hospital, School of Medicine, Shanghai Jiao Tong University, Shanghai, China; 2 Department of Otolaryngology, Changzheng Hospital, Second Military Medical University, Shanghai, China; Sun Yat-sen University Cancer Center, China

## Abstract

**Background and Objectives:**

Studies investigating the association between glutathione S-transferase M1 (*GSTM1*) gene polymorphism and laryngeal cancer risk have reported conflicting results. The aim of the present study was to conduct a meta-analysis assessing the possible associations of *GSTM1* gene polymorphism with laryngeal cancer risk.

**Methods:**

The relevant studies were identified through a search of PubMed, Embase, ISI Web of Knowledge and Chinese National Knowledge Infrastructure until May 2011 and selected on the basis of the established inclusion criteria for publications, then a meta-analysis was performed to quantitatively summarize association of *GSTM1* polymorphism with laryngeal cancer susceptibility.

**Results:**

Seventeen studies were included in the present meta-analysis (2,180 cases and 2,868 controls). The combined results based on all studies showed that *GSTM1* null genotype was associated with increased laryngeal cancer risk (OR = 1.17, 95% CI = 1.04∼1.31). When stratifying for race, *GSTM1* null genotype exhibited increased laryngeal cancer risk in Caucasians (OR = 1.15, 95% CI = 1.01∼1.31), while no significant association was detected in Asians (OR = 1.25, 95% CI = 0.80∼1.96). In the subgroup analysis based on source of controls, significant associations were observed in the population-based studies (OR = 1.15, 95% CI = 1.01∼1.31) yet not in the hospital-based studies (OR = 1.25, 95% CI = 0.93∼1.67). Furthermore, in the subgroup analysis based on sample size, significant associations were also found in studies with at least 50 cases and 50 controls (OR = 1.15, 95% CI = 1.02∼1.30) but not in studies with fewer than 50 cases or 50 controls (OR = 1.46, 95% CI = 0.87∼2.46).

**Conclusions:**

This meta-analysis supported that the *GSTM1* gene polymorphism was associated with laryngeal cancer, particularly in Caucasians, and these associations varied in different subgroup, which indicated that population-based study with larger sample size was more appropriate in design of future study.

## Introduction

Squamous cell carcinoma (SCC) of larynx is the most frequent malignancy in the head and neck region, the risk of which results from complex interactions between many genetic and environmental factors [1]. Numerous epidemiological studies indicate that tobacco smoking and alcohol consumption play a critical role in the development of the disease [2]. Despite many individuals have been exposed to these exogenous risk factors, laryngeal SCC does not develop in all exposed people, which is suggested that susceptibility to cancer might be due to genetic polymorphisms in certain genes that cause differences in the metabolism of carcinogens [3]. Accumulating evidence indicates that genetic polymorphisms have also been extensively investigated to identify inherited genetic risk for laryngeal SCC [4].

Human glutathione S-transferases (*GSTs*), which can be divided into classes of alpha, mu, pi, sigma and theta on the basis of chromosomal location and sequence homology, are a family of cytosolic enzymes that play an important role in the detoxification of potential carcinogens [5]. Among *GSTs* gene family, *GSTM1* gene located on the chromosome 1p13.3, participates in the deactivation of carcinogenic intermediates of polycyclic aromatic hydrocarbons present in tobacco [6], which has been found polymorphic in the population, that is, “present” or “null” genotype may characterize an individual. Interindividual variation in the *GSTM1* genotypes has been investigated in relation to various types of cancers [7].

Many studies have evaluated the potential role of *GSTM1* null genotype in the development of laryngeal SCC. The first study conducted by Jahnke et al. suggested that the *GSTM1* null genotype was not associated with a risk to develop laryngeal cancer [8]. Conversely, the study conducted by Coutelle et al. demonstrated that the risk of laryngeal SCC might be associated with the *GSTM1* null genotype [9]. A series of related studies were carried out later, however, results were generally inconsistent and inconclusive. Concerning the *GSTM1* null genotype in laryngeal cancer, two meta-analyses have been published before 2009, nevertheless, they had not reached unanimity in their conclusions [10], [11]. The earlier meta-analysis by Hashibe et al. did not point to any association of *GSTM1* gene polymorphisms with laryngeal cancer susceptibility [10], whereas the most recent study by Zhuo et al. supported that *GSTM1* deficiency was associated with laryngeal cancer risk [11]. Careful examination of the meta-analysis by Zhuo et al. revealed that three otherwise eligible case-control studies had not been taken into account, on the other hand, only one subgroup analysis on ethnicity was performed, without concerning others such as source of controls and sample size. Therefore, we conducted this updated meta-analysis that might increase statistical power to address the possible associations of *GSTM1* gene polymorphism with laryngeal cancer risk.

## Materials and Methods

### Literature Search Strategy

The meta-analysis process followed the Preferred Reporting Items for Systematic Reviews and Meta-Analyses (PRISMA) guidelines [12] (Checklist S1). Eligible studies published up to May 2011, were identified by a search of PubMed, Embase, ISI Web of Knowledge, and Chinese National Knowledge Infrastructure, with a combination of the following keywords: “Glutathione S-transferase M1,” “*GSTM1*,” “polymorphism,” “larynx,” and “cancer.” The search was done without restriction on language, and was conducted on human subject. All searched papers were retrieved, and their references were checked as well for other relevant publications. Review articles were also searched to find additional eligible studies. For papers on the same population or with overlapping data, only the most recent or the ones with the largest group of subject data-set were included in this analysis. To identify potentially eligible papers, the title and abstract of each paper identified by the literature search were assessed independently by two authors. Disagreements were resolved by discussion.

**Figure 1:**
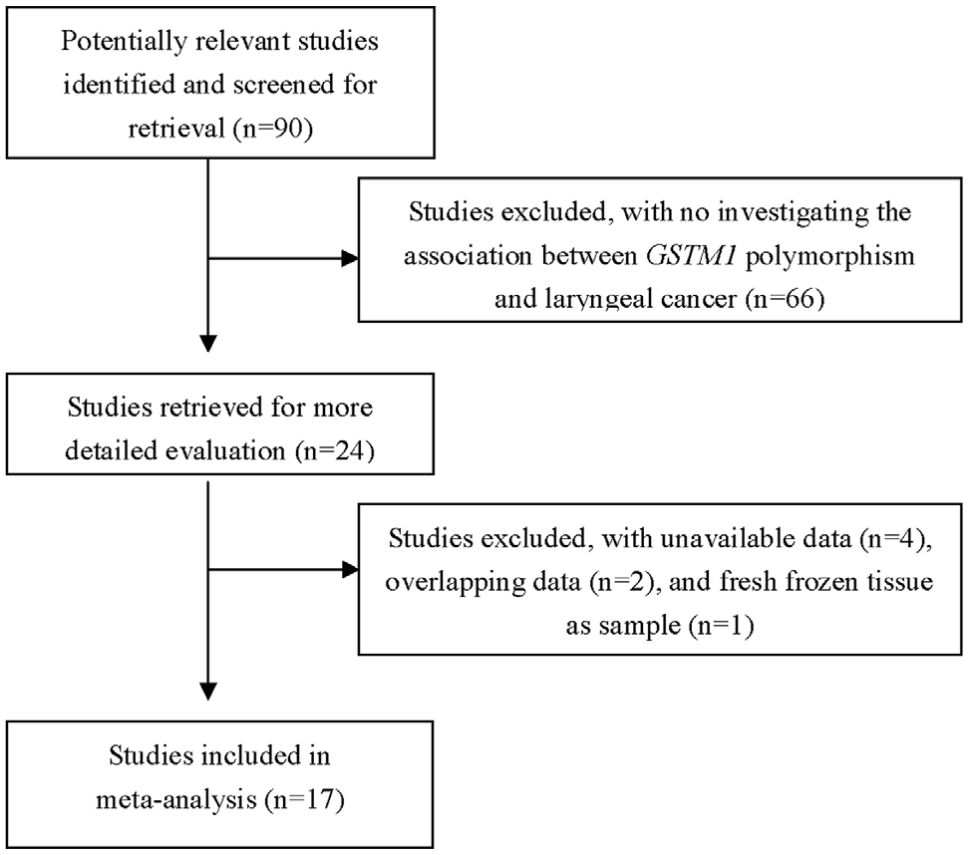
Flow chart of study selection.

**Table 1:** Characteristics of all studies included in the meta-analysis.

Author (Year)	Ethnicity (Country)	Control source	Genotyping method	Cases		Controls	
				Null	Present	Null	Present
Acar 2006	Caucasian (Turkey)	PB	multiplex PCR	57	53	74	123
Bardakci 2003	Caucasian (Turkey)	PB	multiplex PCR	17	19	18	17
Chatzimichalis 2010	Caucasian (Greece)	PB	multiplex PCR	74	14	88	14
Coutelle 1997	Caucasian (France)	PB	PCR	14	4	18	19
Gajecka 2005	Caucasian (Poland)	PB	multiplex PCR	140	152	164	157
Gronau 2003	Caucasian (Germany)	PB	multiplex PCR	39	14	68	71
Hong 2000	Asian (Korea)	PB	multiplex PCR	56	26	33	30
Jahnke 1995	Caucasian (Germany)	PB	PCR	79	58	33	32
Jahnke 1997	Caucasian (Germany)	PB	PCR	151	118	112	104
Jourenkova 1998	Caucasian (France)	HB	multiplex PCR	78	51	90	82
Kihara 1997	Asian (Japan)	PB	multiplex PCR	25	27	230	242
Li 2004	Asian (China)	PB	multiplex PCR	50	39	69	95
Matthias 1998	Caucasian (Germany)	HB	multiplex PCR	151	114	95	83
Morita 1999	Asian (Japan)	PB	PCR	30	39	83	81
Risch 2003	Caucasian (Germany)	PB	multiplex PCR	127	118	135	116
To-Figueras 2002	Caucasian (Spain)	PB	multiplex PCR	96	108	100	103
Unal 2004	Caucasian (Turkey)	PB	PCR	19	23	32	57

Abbreviations: PB, population-based study; HB, hospital-based study; PCR, polymerase chain reaction; RFLP, restriction fragment length polymorphism.

### Inclusion and Exclusion Criteria

All papers involving studies that investigated *GSTM1* polymorphism and laryngeal cancer risk were included. The following inclusion criteria were used for the paper selection: 1) The association of laryngeal cancer with *GSTM1* polymorphisms was clearly evaluated; 2) Only the case-control studies were considered; 3) The paper described the diagnoses of laryngeal cancer and the sources of cases and controls; 4) The polymorphism was determined by polymerase chain reaction (PCR)-based method, using the peripheral blood sample; 5) Enough information about the number of laryngeal cancer cases and controls studied with the different *GSTM1* genotypes were offered. Accordingly, the following exclusion criteria were also used: 1) Study design other than case-control method; 2) Not offering the source of cases and controls and other essential information; 3) Reviews and repeated literature were also excluded.

**Table 2:** Overall and subgroup analysis of *GSTM1* and laryngeal cancer risk.

Meta-analysis groups	Number of studies	Number of Cases/controls	*I* ^2^(%)	OR (95% CI )	Z test *P*value	Begg test*P* value	Egger test *P* value
Overall analysis	17	2180/2868	44	1.17(1.04, 1.31)	0.01	0.11	0.06
Ethnicity							
Caucasian	13	1888/2005	43	1.15(1.01, 1.31)	0.03	0.08	0.07
Asian	4	292/863	58	1.25(0.80, 1.96)	0.34	1.00	0.81
Source of controls							
Population	15	1786/2518	50	1.15 (1.01, 1.31)	0.03	0.11	0.07
Hospital	2	394/350	0	1.25(0.93, 1.67)	0.14	1.00	–
Sample size							
≥50 cases and ≥50 controls	14	2084/2707	47	1.15 (1.02, 1.30)	0.02	0.10	0.09
<50 cases or <50 controls	3	96/161	40	1.46 (0.87, 2.46)	0.15	1.00	0.61

**Figure 2:**
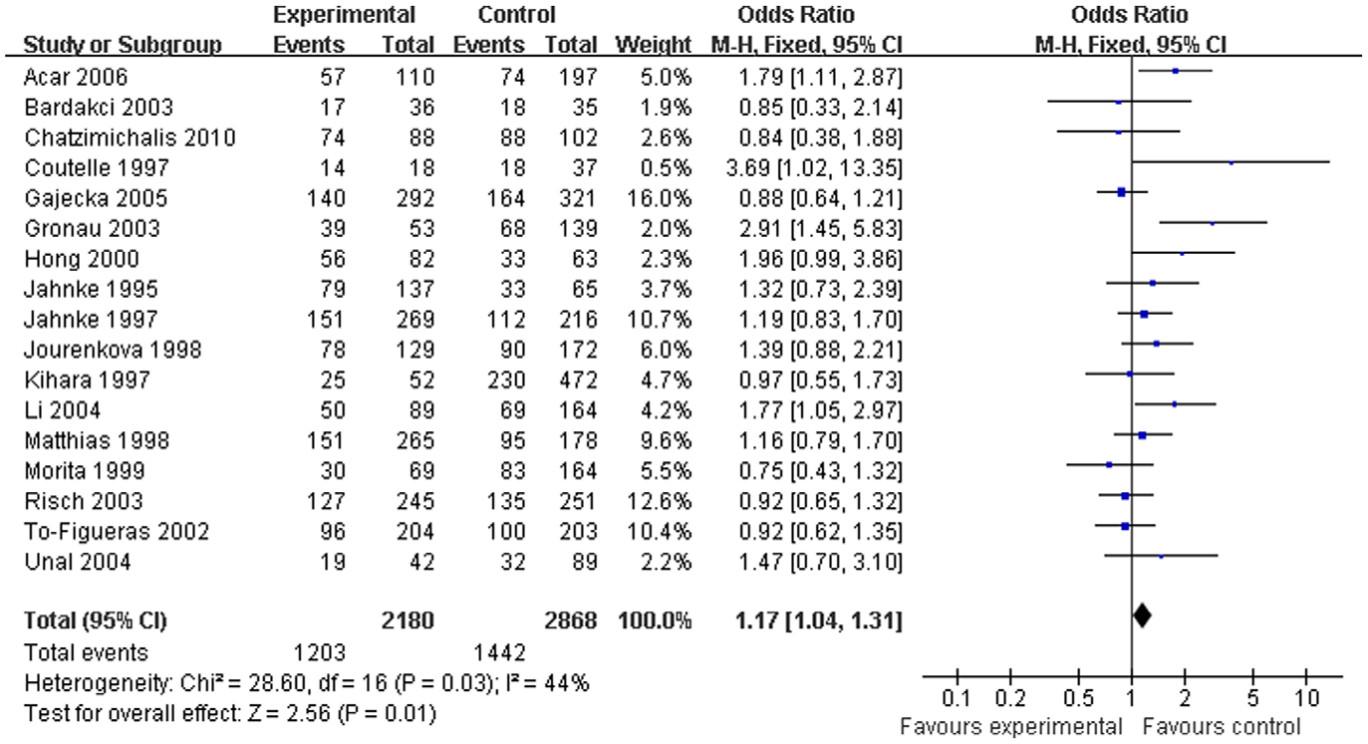
Overall association between *GSTM1* null genotype and laryngeal cancer risk for all subjects (fixed effects).

**Figure 3:**
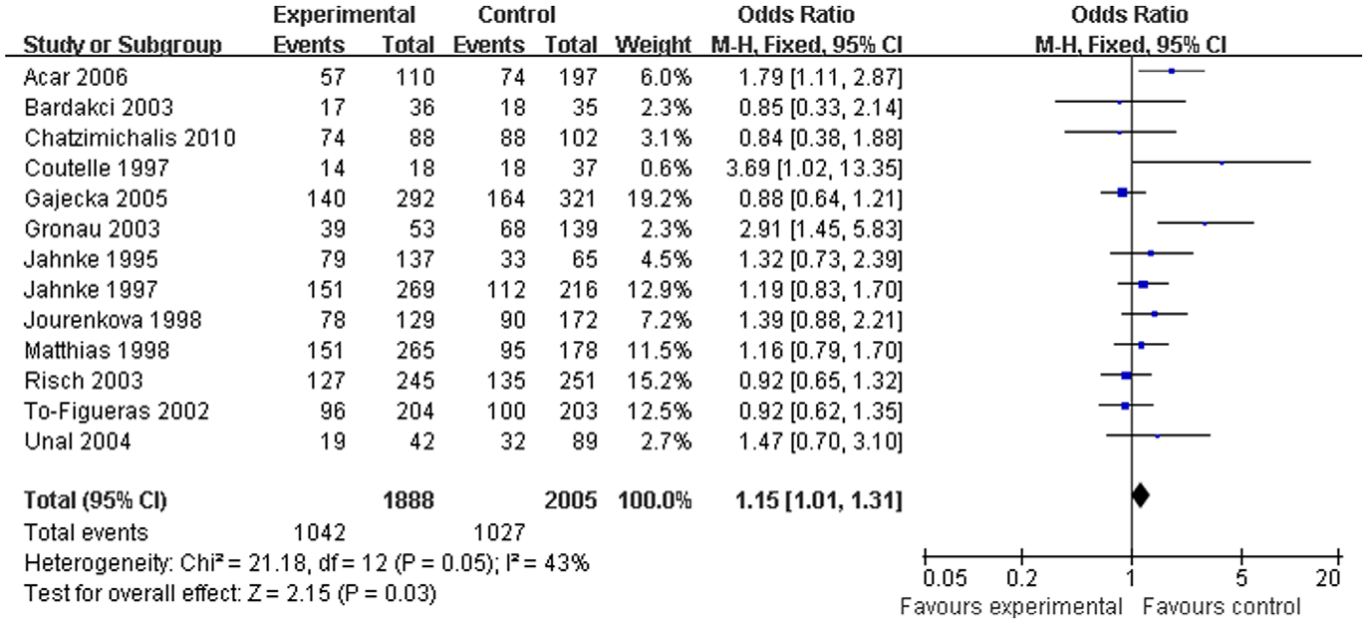
Association between *GSTM1* null genotype and laryngeal cancer risk for Caucasian subjects (fixed-effects).

**Figure 4:**
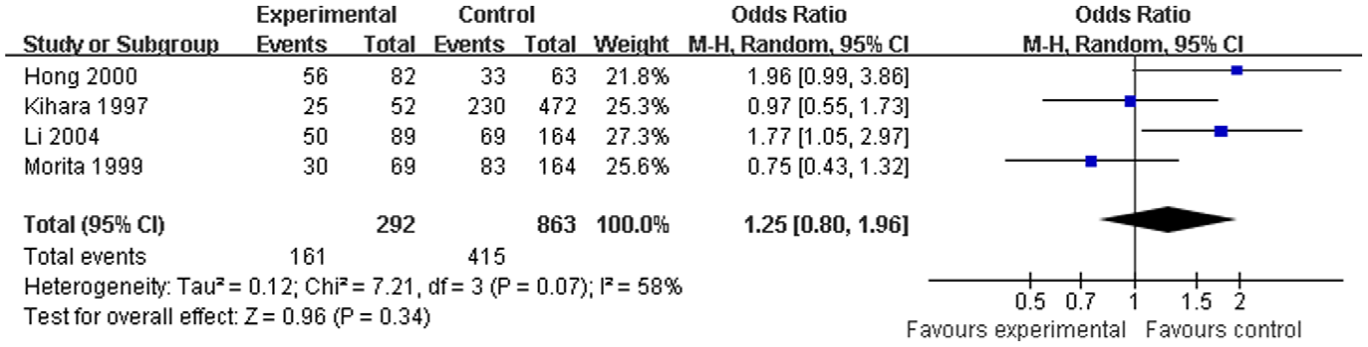
Association between *GSTM1* null genotype and laryngeal cancer risk for Asian subjects (random-effects).

### Data Extraction

From each of the eligible papers, the following data were extracted: first author’s surname, publication year, country of origin, ethnicity, source of controls, genotyping methods, and numbers of different genotypes of cases and controls, respectively. Data were extracted independently by the same authors previously mentioned, and consensuses were reached on all items. Discrepancies were resolved by discussion.

**Figure 5:**
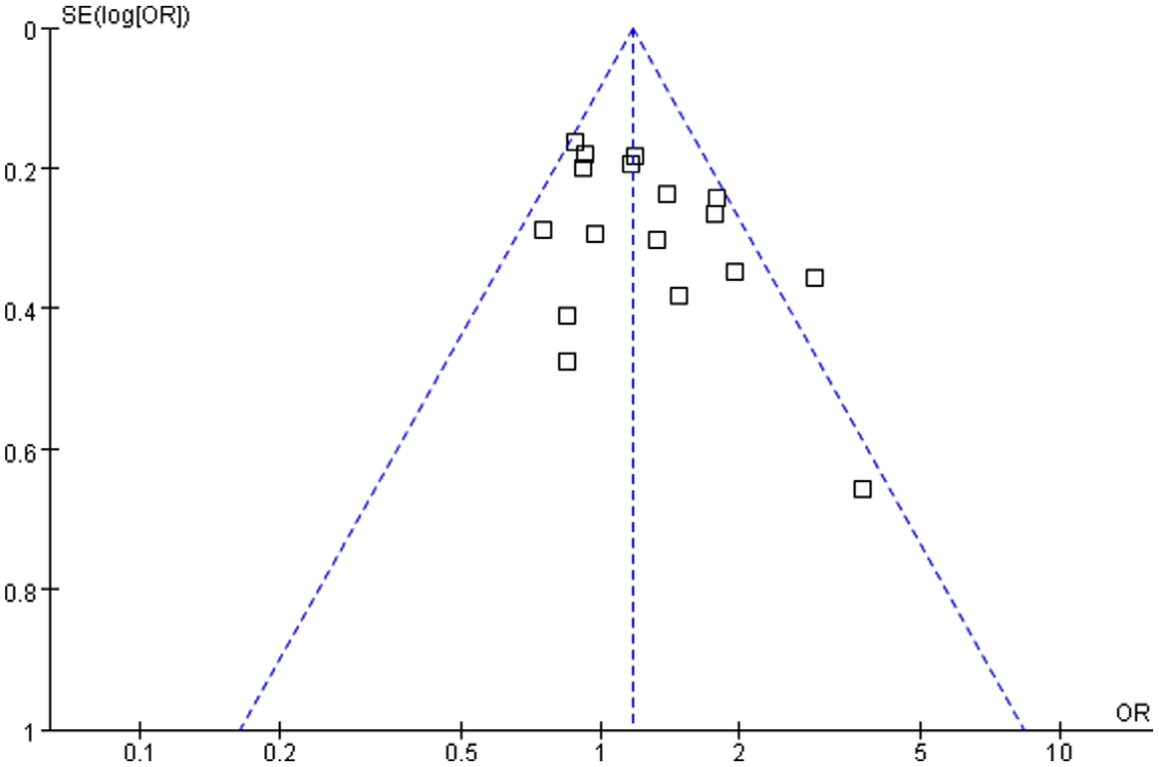
Funnel plot of association between *GSTM1* polymorphism and laryngeal cancer risk.

### Statistical Analysis

The strength of the associations between laryngeal cancer and the *GSTM1* polymorphism was estimated by odds ratio (OR) and 95% confidence interval (CI), based on the genotype frequencies in cases and controls. To calculate the pooled OR, the fixed-effect model and the random-effect model were used. The significance of the pooled OR was determined by the Z-test and *P*<0.05 was considered as statistically significant. Heterogeneity was tested by the *I^2^* statistic [13]. The analyses were also conducted on the subgroups of studies based on ethnicity (Caucasian versus Asian), source of controls (population versus hospital based) and sample size (number of cases and controls ≥50 versus number of cases or controls <50). Visual inspection of asymmetry in funnel plots was used to estimate the potential publication bias [14]. In order to supplement the funnel plot, Begg’s funnel plots [15] and Egger’s regression method [16] were performed (*P*<0.05 was considered representative of statistically significant publication bias). Sensitivity analysis was conducted by removing each individual study in turn from the total and re-analyzing the remainder. Statistical analysis was done using the program Review Manager 5.1.2 (2011, The Cochrane Collaboration) and the statistical software Stata10.1 (Stata Corporation, College Station, TX).

## Results

### Study Characteristics

The literature search identified 90 related articles through PubMed, Embase, ISI Web of Knowledge, and Chinese National Knowledge Infrastructure. With the step of screening the title or abstract, 24 eligible studies were selected (Figure 1). Of the 24 articles selected, 4 studies were excluded because of the lack of data about *GSTM1* status of laryngeal cancer [4], [17], [18], [19], 2 studies [20], [21] were excluded by reason of their data overlapped with that of other two studies respectively [22], [23], study by Hanna et al. [24]was excluded because polymorphism was determined in fresh frozen tissue specimens. Finally, 17 studies including 2,180 laryngeal SCC cases and 2,868 controls [8], [9], [22], [23], [25], [26], [27], [28], [29], [30], [31], [32], [33], [34], [35], [36], [37], were included in this meta-analysis based on inclusion and exclusion criteria (Table 1). Thirteen of the studies were conducted in Caucasians, and four in Asians. Population-based controls were used in 15 studies and hospital-based controls were used in 2 studies. There were 14 studies with larger sample size (number of cases and controls ≥50) and 3 studies with small sample size (number of cases or controls <50). Almost all of the cases were pathologically confirmed.

### Test of Heterogeneity

No heterogeneity was observed in the analysis of hospital-based study (*I^2^* = 0). Moderate heterogeneity was detected in the analysis of Caucasian population (*I^2^* = 43%), population-based study (*I^2^* = 50%), the studies stratified by sample size (large-sample study, *I^2^* = 47%; small-sample study, *I^2^* = 40%) and the overall studies (*I^2^* = 44%). High heterogeneity was found in the analysis of Asian population (*I^2^* = 58%). A random-effect model was used in the analysis of Asian population, and fixed-effect models were selected in other analyses.

### Quantitative Data Synthesis

Both the overall analysis and the subgroup analysis based on ethnicity, source of controls, and sample size were performed (Table 2). The combined results based on all studies showed that the *GSTM1* null genotype was associated with increased laryngeal cancer risk (OR = 1.17, 95% CI = 1.04∼1.31, Figure 2). When stratifying for race, the pooled ORs for *GSTM1* null genotype were 1.15 (95% CI = 1.01∼1.31, Figure 3) in Caucasians and 1.25 (95% CI = 0.80∼1.96, Figure 4) in Asians, which indicated that the significant association confined to Caucasians but not Asians. In the subgroup analysis based on source of controls, significant associations were observed in the population-based studies (OR = 1.15, 95% CI = 1.01∼1.31) yet not in the hospital-based studies (OR = 1.25, 95% CI = 0.93∼1.67). Furthermore, significant associations were also found in studies with large sample size (OR = 1.15, 95% CI = 1.02∼1.30) but not in studies with small sample size (OR = 1.46, 95% CI = 0.87∼2.46).

### Sensitivity Analysis and Publication Bias

A single study involved in the meta-analysis was deleted each time to reflect the influence of the individual data-set to the pooled ORs, and the corresponding pooled ORs were not materially altered (data not shown), indicating that our results were statistically robust. In order to compare the difference and evaluate the sensitivity of the meta-analyses, we also reported the results of the random effects model, the combined OR was 1.21 (95% CI = 1.03∼1.44), similar to the results of the fixed effects model.

Funnel plots were created to assess the possible publication biases, the shape of which did not reveal any evidence of obvious asymmetry (Figure 5). Begg’s funnel plot indicated no evidence for funnel plot asymmetry in either overall analysis (*P* = 0.11) or subgroup analyses. Egger’s test also suggested the absence of publication bias in either overall analysis (*P* = 0.06) or subgroup analyses.

## Discussion

A series of studies have indicated that *GSTM1* gene polymorphism may contribute to risk of laryngeal SCC. However, the results of these studies assessing the association between laryngeal SCC susceptibility and *GSTM1* genotype have been contradictory. Hence, we undertook this meta-analysis, which supported that *GSTM1* gene polymorphism was associated with laryngeal cancer and suggested that *GSTM1* null genotype had an effect on the risk of developing laryngeal cancer among Caucasians.

As one of the most important phase II enzymes, *GSTM1* was known to abolish enzyme activities and therefore has been linked with an increase in a number of cancers, most likely due to increased susceptibilities to environmental toxins and carcinogens. Previous meta-analyses suggest that *GSTM1* null genotypes might have a correlation with increased susceptibilities to breast cancer [38] and lung cancer in Chinese people [7], [39]. In contrast, a number of meta-analyses indicate no marked associations of *GSTM1* gene polymorphism with hepatocellular carcinoma [40], gastric cancer [41], esophageal cancer [42] and prostate cancer [43]. The present meta-analysis, which included 2,180 cases of laryngeal SCC and 2,868 controls, maintained that there was association between *GSTM1* gene polymorphism and laryngeal SCC susceptibility. The combined results based on all studies showed that *GSTM1* null genotype was associated with increased laryngeal cancer risk (OR = 1.17, 95% CI = 1.04∼1.31). Considering the variation of ethnic populations, we further utilized subgroup analysis with respect to ethnicity. When stratifying for race, *GSTM1* null genotype exhibited increased laryngeal cancer risk in Caucasian populations (OR = 1.15, 95% CI = 1.01∼1.31), lack of significant association was detected in Asian populations (OR = 1.25, 95% CI = 0.80∼1.96), suggesting a possible role of ethnic differences in genetic backgrounds and the environment they live in. On the other hand, it is likely that the observed ethnic differences may be due to chance because small number of studies among Asians may lead to insufficient statistical power to detect a slight effect.

The subgroup analysis based on source of controls also suggested that the *GSTM1* null genotype was associated with laryngeal cancer risk in the population-based studies, which supported the association between *GSTM1* polymorphism and laryngeal cancer risk. However, in the hospital-based studies, no significant association was detected. Since it is conceivable that such controls may just represent a sample of ill-defined reference population, and may not be representative of the general population very well, particularly when the genotypes under investigation were associated with the disease conditions that the hospital-based controls may have. In the subgroup analysis by sample size, the pooled OR in studies with larger sample size (number of cases and controls ≥50) was approximately the same in all the studies, which indicated that larger sample size with adequate power was one of the important factors in the design of case-control studies. The use of population-based studies with larger sample size is, therefore, more appropriate in such genetic association studies. It was worth mentioning that power was an issue as limited numbers were in the Asian, small study, and hospital-based groups, therefore, the case-control data were not enough to strongly support our meta-analysis result in these subgroups, which must be interpreted with caution.

Several limitations of this study should be addressed. First, the sample size was still relatively small for some stratified analyses. Second, only published studies were included in the meta-analysis, therefore, publication bias may have occurred, even though the use of a statistical test did not show it. Third, we were unable to obtain information from most studies on the presence or absence of a history of smoking, because of lack of the investigation of gene-environment interactions. Finally, our meta-analysis was based on unadjusted estimates, whereas a more precise analysis could be performed if individual data were available and would allow for an adjustment estimate. Despite the limitation, our meta-analysis significantly increased the statistical power of the analysis based on substantial number of cases and controls from different studies.

In conclusion, this meta-analysis supported that the *GSTM1* gene polymorphism was associated with laryngeal cancer, particularly in Caucasians. As studies among other ethnic populations are currently limited, it is of great essentiality to conduct large-sample studies with wider spectrum of subjects to investigate the relationship between *GSTM1* gene polymorphism and laryngeal SCC risk, which would greatly help summarize the results from published papers.

## Supporting Information

Checklist S1 (DOC)
